# Antimicrobial combination effects against multidrug‐resistant *Acinetobacter baumannii* and *Pseudomonas aeruginosa* strains: A cross‐sectional study

**DOI:** 10.1002/hsr2.2061

**Published:** 2024-04-29

**Authors:** Hossein Kazemian, Morteza Karami‐Zarandi, Hamid Heidari, Roya Ghanavati, Saeed Khoshnood

**Affiliations:** ^1^ Clinical Microbiology Research Center Ilam University of Medical Sciences Ilam Iran; ^2^ Department of Microbiology, Faculty of Medicine Ilam University of Medical Sciences Ilam Iran; ^3^ Department of Microbiology, Faculty of Medicine Zanjan University of Medical Sciences Zanjan Iran; ^4^ Department of Microbiology, Faculty of Medicine Shahid Sadoughi University of Medical Sciences Yazd Iran; ^5^ School of Paramedical Sciences Behbahan Faculty of Medical Sciences Behbahan Iran

**Keywords:** *Acinetobacter baumannii*, drug resistance, drug synergism, *Pseudomonas aeruginosa*

## Abstract

**Background and Aims:**

Emergence of multidrug resistance in non‐fermenting Gram‐negative bacilli is a threat to public health. Combination therapy is a strategy for the treatment of antibiotic‐resistant infections.

**Methods:**

In this cross‐sectional study, a total of 63 nonduplicate clinical isolates of *Acinetobacter baumannii* and *Pseudomonas aeruginosa* were collected from various specimens. Identification of bacterial isolates was performed by phenotypic and molecular tests. Antibiotic susceptibility patterns and detection of β‐lactamase genes were determined using the broth microdilution and polymerase chain reaction (PCR) techniques, respectively. Then, the combined effects analysis was determined by the checkerboard method. Based on the status of resistance to carbapenems (imipenem and meropenem), 25 isolates of each genus were selected for further investigation.

**Results:**

For *A. baumannii, bla*_OXA‐23_, *bla*_OXA‐58_, and *bla*_OXA‐48_ genes were positive in 21 (84%), 17 (68%), and 11 (44%) of isolates, respectively. In *P. aeruginosa* isolates, *bla*_VIM_ was the most common gene (44%) and other genes including *bla*_IMP_, *bla*_NDM_, and *bla*_OXA‐23_ were found in nine (36%), six (24%), and three (12%) isolates, respectively. Meropenem (MER)‐tigecycline (TIG) had a significant synergistic effect against 20 (80%) *A. baumannii* (*p* value < 0.001). This combination was also efficient against 5 (20%) *P. aeruginosa* isolates. Moreover, the other combination, tigecycline‐amikacin (TIG‐AMK) was effective against 10 (40%) *A. baumannii* isolates. The combination of colistin (COL) and MER showed a significant synergistic effect against 21 (84%) *A. baumannii* (*p* value < 0.001) and 17 (68%) *P. aeruginosa* isolates (*p* value < 0.001).

**Conclusion:**

The MER‐TIG and COL‐MER combinations are promising options against resistant bacteria. Our study could be helpful for the development of a new treatment recommendation.

## 1 INTRODUCTION

*Acinetobacter baumannii* and *Pseudomonas aeruginosa* are Gram‐negative microorganisms and account for opportunistic infections in immune‐compromised patients.^1^, ^2^ *P. aeruginosa* produces numerous virulence factors and is responsible for wound and pulmonary infections in burn or Chronic obstructive pulmonary disease patients.^3^ On the other hand, *A. baumannii* poses intrinsic antibiotic resistance and some virulence factors and accounts for various infections such as ventilator‐associated pneumonia, and wound and catheter infections.^1^, ^4^ However, antibiotic resistance is the common feature of these organisms that intensifies the infection treatment and outcome. Antibiotic resistance arises intrinsically, and horizontal gene transfer and mutations frequently happen in these two opportunistic pathogens and escalate antibiotic resistance. During the last decades, β‐lactam antibiotics especially carbapenems have been used to treat related infections.^3^

Unfortunately, these organisms show high levels of resistance to β‐lactam antibiotics and almost all of the existing antibiotics. In a manner that colistin, tigecycline, and piperacillin‐tazobactam resistance have been reported all over the globe. Antibacterial resistance is a global health problem requiring the development of new therapeutics. Combination therapy by confirmed antibiotics with inhibitors targeting innate resistance mechanisms provides an alternative approach to develop therapeutics.^4^, ^5^ Several studies highlighted the potency of antibiotic combination therapy for defeating multidrug‐resistant (MDR) or extensively drug‐resistant (XDR) isolates of *A. baumannii* and *P. aeruginosa*.^6^ New combinations are increasingly suggested as a choice of therapeutic with the increasing approval of new drugs. In a world where new antibiotics discovery and production are far slower than the development of antibiotic resistance in human pathogens, although temporarily, combination therapy could be an opportunity to overcome the threat of MDR and XDR nosocomial infection.^7^

Potential benefits with combinations as compared with monotherapy include synergistic effects, a broader antibacterial spectrum, and reduced risk for developing drug resistance. In the absence of evidence‐based treatment choices, combinations are employed to increase the antibacterial effects of available antibiotics against MDR bacterial isolates.^8^ Simultaneous exposure of drug‐resistant bacteria to antibiotics in combination therapy improves the efficacy of antibiotics by several mechanisms.^7^ For instance, cell membrane damaging antibiotics like colistin result in the higher entrance of β‐lactams or tetracycline antibiotics inside the cells or blocking destructive enzyme functions such as β‐lactamases recover the activity of β‐lactam antibiotics.^9^ In the current study, we tried to assess the efficacy of different antibiotic combinations and introduce potential combinations for the treatment of antibiotic‐resistant strains.

## 2 MATERIALS AND METHODS

### 2.1 Bacterial isolates

This cross‐sectional study received ethical approval from the local ethics committee of Behbahan University of Medical Sciences (IR.BHN.REC.1401.014). During 2020 (the beginning of March to the end of October), various clinical samples (catheter, sputum, trachea, urine, bronchoalveolar lavage [BAL], blood, and wound) were collected from Shahid Motahari and Imam Khomeini hospitals, Tehran, Iran. Samples were inoculated on blood agar and MacConkey agar (Merck). Primary identification *A. baumannii* isolates was based on the colony morphology and Gram staining reaction. Standard biochemical tests such as oxidation and fermentation (OF) of sugars, methyl red, Voges Prausker, triple sugar iron (TSI) agar, urease test, catalase, oxidase, citrate, and indole production were used to identify the *A. baumannii* isolates. *P. aeruginosa* isolates were identified by conventional biochemical tests, including Gram stain, catalase, oxidase, TSI, ability of growth in 42°C, and OF test.^10^ Of 156 positive cultures, 63 positive cultures were detected as *A. baumannii* (*n* = 27) or *P. aeruginosa* (*n* = 36) isolates. Molecular identification of *A. baumannii* and *P. aeruginosa* was also performed by PCR method using special primers for *bla*_oxa‐51_ and *lasR* genes, respectively. The *A. baumannii* ATCC 19606 and *P. aeruginosa* ATCC 27853 were used as positive control strains.

### 2.2 Antibiotic susceptibility testing

Antibiotic susceptibility patterns of the isolates were determined by broth microdilution method according to CLSI (Clinical and Laboratory Standards Institute) criteria. Minimum inhibitory concentration (MIC) of selected antibiotics including meropenem (MER), imipenem (IMI), ciprofloxacin (CIP), colistin (COL), amikacin (AMK), tigecycline (TIG), ceftazidime (CAZ), piperacillin‐tazobactam (PIP/TAZ) were determined, and based on the status of resistance to carbapenems (imipenem and meropenem), 25 isolates of each genus were selected for further investigations. The MIC was determined in triplicate for each isolate. In *A. baumannii* isolates, the MICs for Tigecycline, were evaluated according to the criteria suggested by Jones et al. (MIC ≥ 8 μg/mL, as resistant).^11^ In *P. aeruginosa* isolates, the MICs for this antibiotic were assessed in accordance with the European Committee on Antimicrobial Susceptibility Testing (EUCAST) criteria (MICs of 1 μg/mL is susceptible, 2 μg/mL is intermediate, and >2 μg/mL is resistant). Multidrug‐resistant *A. baumannii* and *P. aeruginosa* (MDR‐AB or MDR‐PA) were classified as strains that were resistant to at least one agent in three different antimicrobial classes such as carbapenems, cephalosporins, and aminoglycosides. The XDR isolates (XDR‐AB or XDR‐PA) were defined as strains that were resistant to at least one agent in all but ≤2 antimicrobial categories.^6^

### 2.3 Detection of carbapenemase genes

Genomic DNA from 25 *A. baumannii* and 25 *P. aeruginosa* isolates were extracted using the genomic DNA extraction kit (GeneAll) according to the manufacturer's protocol. Using specific primers (Table 1), the existence of β‐lactamase genes (*bla*_IMP_, *bla*_KPC_, *bla*_NDM_, *bla*_VIM_, *bla*_OXA‐23_, *bla*_OXA‐48_, and *bla*_OXA‐58_) was detected with polymerase chain reaction (PCR). The PCR tests were performed in a final volume of 25 μL containing 3 μL of extracted DNA, 200 μM each dNTP, 10 pmol of each primer, and 1.5 U of *Taq* DNA polymerase in 1 × PCR buffer containing 1.5 mM MgCl_2_ (Qiagen). PCR reactions were run using a SuperCycler Thermal Cycler (Kyratec), and the PCR products were subjected to 1.5% agarose gel electrophoresis.

**Table 1:** Primers used in this study.

Gene	Sequence (5′−3′)	Reference
*bla*_IMP_	F: GTTAACGGGTGGGGCGTTG	12
	R: AGCCACTCTATTCCGCCCGT	
*bla*_KPC_	F: GTATCGCCGTCTAGTTCTGC	13
	R: GGTCGTGTTTCCCTTTAGCC	
*bla*_NDM_	F: CAGCGCAGCTTGTCG	12
	R: TCGCGAAGCTGAGCA	
*bla*_VIM_	F: TAGCGGTGAGTATCCGACAGT	12
	R: TGCTTCCGGGTAGTGTTGTTG	
*bla*_OXA‐23_	F: CCTCAGGTGTGCTGGTTATTCA	14
	R: CTCCAATCCGATCAGGGCAT	
*bla*_OXA‐48_	F: CTGGTGGGTYGGTTGGGTTGA	15
	R: GCAGCCCTAAACCATCCGATGT	
*bla*_OXA‐58_	F: GTTGTATGTAGAGCGCAGAGG	14
	R: ACCCACATACCAACCCACTTG	

### 2.4 Synergistic effect analysis

Synergy of MER‐AMK, MER‐TIG, TIG‐AMK, COL‐MER, and IMI‐MER was assessed using checkerboard method. In short, increasing concentrations of each couple of antibiotics were prepared in each column and raw of different 96‐well microtiter plates. Then, a suspension containing 5 × 10^5^ CFU/mL was prepared for each isolate in wells. Synergistic effect was determined in triplicate for each isolate. The fractional inhibitory concentration index (FICI) was calculated using the following equation.^16^ FICI= (MIC_A+C_/MIC_A_) + (MIC_B+C_/MIC_B_). The FICI interpretation criteria were as follows: synergy: FICI ≤ 0.5; indifference: 0.5 < FICI < 4.0; and antagonism: FICI > 4.0.

### 2.5 Statistical analysis

Statistical analysis was performed using the “IBM SPSS Statistics 22” software (IBM Analytics). Statistical significance of variables determined by chi‐square test. The results are presented as descriptive statistics in terms of relative frequency. A *p* value of ≤0.05 was considered statistically significant.

## 3 RESULTS

### 3.1 Studied population and bacterial isolates

In the current study, 63 nonduplicative *P. aeruginosa and A. baumannii* isolates were collected from 36 (57.14%) males and 27 (42.86%) females with the mean age of 39.2 ± 7 (range 5–72) years, and with the maximum number of cases in the age group of 40–61 years. These isolates were obtained from different clinical specimens, including blood (3.18%), catheter (9. 52%), sputum (33.33%), urine (3.18%), trachea (7.94%), BAL (19.05%), and wound (23.8%). All 63 isolates originated from the following wards: (24) from intensive care unit (ICU), as well as (13) from surgery, (10) from burn, (6) from operation room, (7) from cardiac care unit (CCU), and (3) from emergency.

### 3.2 Antibiotic resistance patterns

The susceptibility patterns are shown in Table 2. In *A. baumannii* isolates, complete resistance to meropenem, imipenem, amikacin, and ciprofloxacin was observed. The results showed that tigecycline and colistin with susceptibility rates of 100% (MICs range from 0.25 to 4 μg/mL) and 96% (MICs range from 0.5 to 2 μg/mL) were the most efficient agents against *A. baumannii* isolates, respectively. All *Pseudomonas* isolates were also resistant to meropenem, imipenem, and ciprofloxacin. For *A. baumannii*, 16 isolates were MDR and 9 isolates were XDR. In addition 21 MDR, 3 XDR and 1 non‐MDR isolates of *P. aeruginosa* were determined. Among *A. baumannii* and *P. aeruginosa* isolates highest resistance was detected for carbapenem antibiotics such as imipenem and meropenem.

**Table 2:** Antibiotic susceptibility pattern among 50 selected isolates.

*A*. *baumannii (25)*	Susceptibility pattern	*N* (%)									
		R	PIP/TAZ		CAZ	AMK	CIP	IMI	MER	TIG	COL
			13 (52)		21 (84)	100 (100)	25 (100)	25 (100)	25 (100)	0 (0.0)	0 (0.0)
		I	11 (44)		3 (12)	0 (0.0)	0 (0.0)	0 (0.0)	0 (0.0)	0 (0.0)	1 (4)
		S	1 (4)		1 (4)	0 (0.0)	0 (0.0)	0 (0.0)	0 (0.0)	25 (100)	24 (96)
	Interpretive categories and MIC breakpoints (µg/mL)	R	≥128/4		≥32	≥16	≥4	≥8	≥8	≥ 8	≥4
		I	32/4‐64/4		16	8	2	4	4	‐	≤2
		S	≤16/4		≤8	≤4	≤1	≤2	≤2	‐	‐
*P*. *aeruginosa (25)*	Susceptibility pattern	*N* (%)									
		R		14 (56)	21 (84)	18 (72)	25 (100)	25 (100)	25 (100)	23 (92.0)	0 (0.0)
		I		11 (44)	3 (12)	6 (24)	0 (0.0)	0 (0.0)	0 (0.0)	2 (8.0)	25 (100)
		S		0 (0.0)	1 (4)	1 (4)	0 (0.0)	0 (0.0)	0 (0.0)	0 (0.0)	0 (0.0)
	Interpretive categories and MIC breakpoints (µg/mL)	R		≥128/4	≥32	≥64	≥2	≥8	≥8	>2	≥4
		I		32/4–64/4	16	32	1	4	4	2	≤2
		S		≤16/4	≤8	≤16	≤ 0.5	≤2	≤2	1	‐

Abbreviations: AMK, amikacin; CAZ, ceftazidime; CIP, ciprofloxacin; COL, colistin; IMI, imipenem; MER, meropenem; PIP/TAZ, piperacillin–tazobactam; TIG, tigecycline.

### 3.3 Distribution of β‐lactamase genes

In *A. baumannii* isolates *bla*_OXA‐23_, *bla*_OXA‐58_, *bla*_OXA‐48_ genes were the most frequent genes and 21 (84%), 17 (68%), and 11 (44%) isolates were positive, respectively. Other genes, including *bla*_IMP_, *bla*_VIM_, and *bla*_NDM_, were present in four (16%), four (16%), and three (12%) isolates, and *bla*_KPC_ was not found (Table 3). Out of *A. baumannii* isolates, 12 isolates had three β‐lactamase genes, and 2 isolates had four genes, simultaneously. Among *P. aeruginosa* isolates *bla*_VIM_ was the most common gene 11 (44%), and other genes including *bla*_IMP_, *bla*_NDM_, and *bla*_OXA‐23_ were found in 9 (36%), 6 (24%), and 3 (12%) isolates, respectively (figs. 1–6 are shown in the Supporting Information 1). Other genes, such as *bla*_KPC_, *bla*_OXA‐48_, and *bla*_OXA‐58_, were not detected in any isolate (Table 4).

**Table 3:** Source of the isolates, MICs of single antibiotics, and distribution of β‐lactamases genes in *A. baumannii* isolates.

Isolate no.	Sample type	MICs of each antibiotic (µg/mL)								β‐lactamases						
		MER	IMI	CIP	COL	AMK	TIG	CAZ	PIP/TAZ	IMP	NDM	VIM	OXA‐23	OXA‐58	OXA‐48	KPC
AB1	Sputum	32	32	4	1	64	1	32	>128/4	+	‐	‐	+	+	‐	‐
AB2	BAL	32	32	4	0.5	64	1	64	32/4	‐	‐	‐	+	+	+	‐
AB3	BAL	16	32	8	0.5	128	4	16	>128/4	‐	‐	‐	‐	+	‐	‐
AB4	Trachea	64	128	8	0.5	128	4	64	>128/4	‐	+	‐	+	+	+	‐
AB5	Sputum	64	64	8	1	128	4	32	>128/4	‐	‐	+	+	‐	‐	‐
AB6	Wound	32	32	8	1	64	0.5	32	64/4	‐	‐	+	‐	‐	‐	‐
AB7	BAL	32	32	8	1	32	0.5	32	64/4	‐	‐	‐	+	+	+	‐
AB8	Blood	64	64	4	1	64	0.5	32	>128/4	+	‐	‐	+	‐	‐	‐
AB9	Wound	16	32	8	0.5	32	1	64	32/4	‐	‐	‐	+	+	‐	‐
AB10	Urine	32	64	4	0.5	16	1	32	>128/4	‐	‐	‐	‐	+	‐	‐
AB11	Sputum	32	32	8	1	32	0.5	32	32/4	‐	‐	‐	+	+	‐	‐
AB12	BAL	32	32	8	0.5	32	1	64	16/4	‐	‐	‐	+	+	+	‐
AB13	Sputum	64	64	8	1	64	0.5	64	>128/4	‐	‐	‐	+	+	+	‐
AB14	Trachea	128	128	4	1	128	0.5	64	>128/4	+	+	‐	+	‐	‐	‐
AB15	Catheter	64	64	4	0.5	64	1	32	>128/4	‐	‐	‐	+	‐	‐	‐
AB16	Sputum	32	64	8	1	64	1	64	>128/4	‐	‐	‐	+	+	+	‐
AB17	Trachea	64	64	8	1	64	2	32	64/4	‐	‐	‐	+	+	+	‐
AB18	Sputum	16	16	4	0.5	16	2	4	64/4	‐	‐	+	‐	+	+	‐
AB19	Sputum	128	128	8	2	128	4	64	>128/4	+	+	‐	+	‐	‐	‐
AB20	Blood	32	64	8	1	128	2	16	64/4	‐	‐	‐	+	‐	‐	‐
AB21	Wound	64	64	8	1	128	4	64	>128/4	‐	‐	+	+	+	+	‐
AB22	Trachea	32	16	8	0.5	64	0.5	32	>128/4	‐	‐	‐	+	+	+	‐
AB23	Sputum	32	32	8	1	64	0.25	64	32/4	‐	‐	‐	+	+	‐	‐
AB24	BAL	16	16	8	0.5	32	0.5	32	32/4	‐	‐	‐	+	+	+	‐
AB25	BAL	16	32	8	1	32	0.5	16	32/4	‐	‐	‐	+	‐	‐	‐
Number of positive isolates for each gene										4	3	4	21	17	11	0

Abbreviations: AB, A. baumannii; AMK, amikacin; CAZ, ceftazidime; CIP, ciprofloxacin; COL, colistin; IMI, imipenem; MER, meropenem; PIP/TAZ, piperacillin–tazobactam; TIG, tigecyclin.

**Table 4:** Source of the isolates, MICs of single antibiotics, and distribution of β‐lactamases genes in *P. aeruginosa* isolates.

Isolate no.	Sample type	MICs of each antibiotic (µg/mL)								β‐lactamases						
		MER	IMI	CIP	COL	AMK	TIG	CAZ	PIP/TAZ	IMP	NDM	VIM	OXA‐23	OXA‐58	OXA‐48	KPC
PA1	Wound	16	16	8	0.5	16	4	32	32/4	‐	+	‐	+	‐	‐	‐
PA2	Sputum	16	16	8	0.5	32	4	16	>128/4	‐	‐	‐	‐	‐	‐	‐
PA3	Wound	64	32	8	1	64	8	64	>128/4	+	‐	+	‐	‐	‐	‐
PA4	BAL	16	16	8	0.5	32	4	32	32/4	‐	‐	+	‐	‐	‐	‐
PA5	Sputum	16	32	4	1	64	8	64	32/4	‐	+	+	‐	‐	‐	‐
PA6	Catheter	16	32	8	1	64	8	32	32/4	‐	+	‐	‐	‐	‐	‐
PA7	Wound	32	32	8	1	128	16	32	>128/4	+	‐	‐	‐	‐	‐	‐
PA8	BAL	32	32	8	1	128	4	32	>128/4	‐	‐	‐	+	‐	‐	‐
PA9	Sputum	64	32	8	1	128	4	64	32/4	+	‐	+	‐	‐	‐	‐
PA10	Wound	16	16	8	0.5	32	8	32	>128/4	+	‐	‐	‐	‐	‐	‐
PA11	Wound	32	32	8	1	64	16	32	>128/4	‐	‐	+	‐	‐	‐	‐
PA12	Sputum	16	32	8	1	64	2	32	32/4	‐	‐	‐	‐	‐	‐	‐
PA13	Wound	16	16	8	0.5	32	4	32	32/4	‐	‐	‐	‐	‐	‐	‐
PA14	BAL	16	32	8	1	32	2	32	32/4	‐	+	‐	‐	‐	‐	‐
PA15	Sputum	16	16	8	0.5	32	4	32	>128/4	+	‐	‐	‐	‐	‐	‐
PA16	Wound	16	64	8	1	64	8	32	64/4	‐	+	‐	‐	‐	‐	‐
PA17	Wound	32	32	4	1	64	16	32	64/4	‐	‐	+	‐	‐	‐	‐
PA18	Catheter	64	64	8	2	64	8	64	>128/4	+	‐	+	‐	‐	‐	‐
PA19	Sputum	32	64	8	1	64	16	16	>128/4	‐	‐	‐	‐	‐	‐	‐
PA20	Catheter	32	32	4	1	64	16	32	>128/4	‐	‐	‐	+	‐	‐	‐
PA21	Sputum	64	64	8	1	128	32	16	>128/4	+	+	+	‐	‐	‐	‐
PA22	Sputum	16	32	8	1	64	8	8	>128/4	‐	‐	‐	‐	‐	‐	‐
PA23	BAL	32	32	4	1	64	8	64	64/4	+	‐	+	‐	‐	‐	‐
PA24	Catheter	64	32	8	1	64	4	32	>128/4	‐	‐	+	‐	‐	‐	‐
PA25	Wound	64	32	4	0.5	64	4	64	>128/4	+	‐	+	‐	‐	‐	‐
Number of positive isolates for each gene										9	6	11	3	0	0	0

Abbreviations: AMK, amikacin; CAZ, ceftazidime; CIP, ciprofloxacin; COL, colistin; IMI, imipenem; MER, meropenem; PA, P. aeruginosa; PIP/TAZ, piperacillin–tazobactam; TIG, tigecyclin.

### 3.4 Synergistic effect analysis

Five combinations, including MER‐AMK, MER‐TIG, TIG‐AMK, COL‐MER, and IMI‐MER, were tested in a checkerboard assay. For MER‐AMK combination, all FICI values were higher than 0.5 and there was not a synergistic effect between these two antibiotics. The other combination MER‐TIG had a significant synergistic effect against 20 (80%) *A. baumannii* isolates (*p* value < 0.001) and this combination was efficient against 5 (20%) *P. aeruginosa* isolates. The third combination, TIG‐AMK was effective against 10 (40%) *A. baumannii* isolates and there was no proper synergistic effect for remnant isolates. Moreover, the combination of COL and MER showed a significant synergistic effect against 21 (84%) *A. baumannii* and 17 (68%) *P. aeruginosa* isolates (*p* value < 0.001). Finally, IMI‐MER combination was operative only against three (12%) *A. baumannii* and five (20%) *P. pseudomonas* isolates (Tables 5, 6, 7).

**Table 5:** In vitro evaluation of antimicrobial combinations.

	FICI of the combinations										
	*A. baumannii*					*P. aeruginosa*					
Isolate	MER‐AMK	MER‐TIG	TIG‐AMK	COL‐MER	IMI‐MER	Isolate	MER‐AMK	MER‐TIG	TIG‐AMK	COL‐MER	IMI‐MER
AB1	0.625	0.5	0.625	0.5	1	PA1	0.625	0.5	1	0.5	0.75
AB2	1	0.5	0.625	<0.5	1	PA2	0.75	0.625	1	<0.5	0.5
AB3	1	<0.5	0.625	<0.5	0.5	PA3	1	0.75	1	0.75	1
AB4	1	0.625	0.5	0.5	1	PA4	1	0.75	1	0.5	1
AB5	0.75	0.5	0.625	0.5	1	PA5	0.75	0.75	0.75	0.5	1
AB6	1	0.5	0.5	0.5	0.5	PA6	1	0.5	1.25	0.5	1
AB7	0.75	0.5	0.5	0.5	0.75	PA7	1	0.5	1.25	0.5	1
AB8	0.75	0.5	1	0.625	0.75	PA8	1	0.5	0.75	0.5	0.625
AB9	1.25	0.625	0.5	0.5	1	PA9	1	0.625	1	0.75	1
AB10	1	0.5	0.625	0.5	0.625	PA10	1	0.625	0.75	0.5	1
AB11	1	0.5	0.75	0.5	1	PA11	0.625	0.75	0.625	0.5	1
AB12	1	<0.5	0.75	0.5	1	PA12	1	0.75	1	<0.5	0.5
AB13	1	0.5	0.5	0.5	1	PA13	1	1	1	0.75	0.5
AB14	1	0.625	0.75	0.75	1	PA14	0.75	0.75	0.625	0.625	1
AB15	0.625	<0.5	0.625	<0.5	0.5	PA15	0.75	0.75	0.75	0.5	1
AB16	0.625	<0.5	0.5	0.5	0.75	PA16	1	1	1	0.5	1
AB17	0.625	<0.5	0.5	0.5	1	PA17	1	1	1	0.5	1
AB18	0.75	0.5	0.5	<0.5	1	PA18	1	0.75	1	0.625	1
AB19	1	0.625	0.5	0.625	1	PA19	1	1	0.75	0.5	0.5
AB20	1	0.5	0.625	0.5	0.75	PA20	1	1	1	0.5	1
AB21	1	0.625	1	0.625	1	PA21	1	0.75	1	0.75	1
AB22	1.25	0.5	0.5	0.5	1	PA22	0.625	0.5	1	0.5	0.5
AB23	1	0.5	1	0.5	1	PA23	1	0.75	1.25	0.5	1
AB24	1	0.5	0.75	0.5	1	PA24	1	0.625	0.75	0.75	1
AB25	1.25	0.5	1	<0.5	1	PA25	1	1	1	0.625	1
*N* (%)	0 (0%)	20 (80%)	10 (40%)	21 (84%)	3 (12%)	N (%)	0 (0%)	5 (20%)	0 (0%)	17 (68%)	5 (20%)

Abbreviations: AB, A. baumannii; AMK, amikacin; COL, colistin; IMI, imipenem; MER, meropenem; PA, P. aeruginosa; TIG, tigecycline.

**Table 6:** Antimicrobial combinations against *A. baumannii* isolates.

Isolate	MER	AMK	MER‐AMK	*p* Value	TIG	MER‐TIG	*p* Value	TIG‐AMK	*p* Value	COL	COL‐MER	*p* Value	IMI	IMI‐MER	*p* Value
AB1	R	R	I	0.999	S	S	<0.001	I	0.334	S	S	<0.001	R	I	0.002
AB2	R	R	I		S	S		I		S	S		R	I	
AB3	R	R	I		S	S		I		S	S		R	I	
AB4	R	R	I		S	I		S		S	S		R	S	
AB5	R	R	I		S	S		S		S	S		R	I	
AB6	R	R	I		S	S		S		S	S		R	I	
AB7	R	R	I		S	S		S		S	S		R	I	
AB8	R	R	I		S	S		I		S	I		R	I	
AB9	R	R	I		S	I		S		S	S		R	I	
AB10	R	R	I		S	S		I		S	S		R	I	
AB11	R	R	I		S	S		I		S	S		R	I	
AB12	R	R	I		S	S		I		S	S		R	I	
AB13	R	R	I		S	S		S		S	S		R	I	
AB14	R	R	I		S	I		I		S	I		R	I	
AB15	R	R	I		S	S		I		I	S		R	S	
AB16	R	R	I		S	S		S		S	S		R	S	
AB17	R	R	I		S	S		I		S	S		R	I	
AB18	R	R	I		S	S		S		S	S		R	I	
AB19	R	R	I		S	I		S		I	I		R	I	
AB20	R	R	I		S	S		I		S	S		R	I	
AB21	R	R	I		S	I		I		S	I		R	I	
AB22	R	R	I		S	S		S		S	S		R	I	
AB23	R	R	I		S	S		I		S	S		R	I	
AB24	R	R	I		S	S		I		S	S		R	I	
AB25	R	R	I		S	S		I		S	S		R	I	

**Table 7:** Antimicrobial combinations against *P. aeruginosa* isolate.

Isolates	MER	AMK	MER‐AMK	*p* Value	TIG	MER‐TIG	*p* Value	TIG‐AMK	*p* Value	COL	COL‐MER	*p* Value	IMI	IMI‐MER	*p* Value
PA1	R	S	I	0.999	R	S	0.051	I	0.999	I	S	<0.001	R	I	0.051
PA2	R	I	I		R	I		I		I	S		R	S	
PA3	R	R	I		R	I		I		I	I		R	I	
PA4	R	I	I		R	I		I		I	S		R	I	
PA5	R	R	I		R	I		I		I	S		R	I	
PA6	R	R	I		R	S		I		I	S		R	I	
PA7	R	R	I		R	S		I		I	S		R	I	
PA8	R	R	I		R	S		I		I	S		R	I	
PA9	R	R	I		R	I		I		I	I		R	I	
PA10	R	I	I		R	I		I		I	S		R	I	
PA11	R	R	I		R	I		I		I	S		R	I	
PA12	R	R	I		I	I		I		I	S		R	S	
PA13	R	I	I		R	I		I		I	I		R	S	
PA14	R	I	I		I	I		I		I	I		R	I	
PA15	R	I	I		R	I		I		I	S		R	I	
PA16	R	R	I		R	I		I		I	S		R	I	
PA17	R	R	I		R	I		I		I	S		R	I	
PA18	R	R	I		R	I		I		I	I		R	I	
PA19	R	R	I		R	I		I		I	S		R	S	
PA20	R	R	I		R	I		I		I	S		R	I	
PA21	R	R	I		R	I		I		I	I		R	I	
PA22	R	R	I		R	S		I		I	S		R	S	
PA23	R	R	I		R	I		I		I	S		R	I	
PA24	R	R	I		R	I		I		I	I		R	I	
PA25	R	R	I		R	I		I		I	I		R	I	

## 4 DISCUSSION

It is crucial to assess the effects of combination therapy on the evolution of antibiotic resistance and the fitness of bacteria in comparison to monotherapy. This evaluation holds significance from both an evolutionary and medical standpoint. Combination therapy has been proposed as a potential strategy to reduce the rate at which antibiotic resistance develops. However, there have been limited experimental studies on combination therapy, and the available data on its beneficial effects are inconsistent. In the present study, most of the isolates harbored at least one of the aforementioned β‐lactamases, and except for five *P. aeruginosa* isolates, the remnant was positive. Different reports from all over the globe have suggested the presence of these genes among *A. baumannii* and *P. aeruginosa* isolates.^12^, ^13^, ^17^, ^18^ A major reason for the high levels of resistance to carbapenem antibiotics is the emergence of broad‐spectrum‐ Extended Spectrum Beta‐Lactamase (ESBL), such as *KPC, OXA, IMP*, VIM, and *NDM*. However, the prevalence of ESBL producing bacteria varied among different studies, which may be associated with the differences in the geographical area, type of infection, and settings. The highest carbapenem MICs were observed in the isolates harboring at least one β‐lactamase gene. On the other hand, five isolates without any detected β‐lactamases had relatively lower MICs. The presence of these genes not only limits the efficacy of the carbapenem antibiotics but also is a barrier to the high synergistic effect of the other antibiotics and carbapenems. So, the higher MICs of carbapenems can related to weaker synergistic effects among COL, TIG, MER, and IMI. According to the antibiotic susceptibility testing, all the isolates of *A. baumannii* and *P. aeruginosa* were sensitive to COL, which is consistent with the interhospital monitoring results in our hospital. In contrast to our study, Elham et al. reported 8.5% colistin resistance among 136 *Acinetobacter* isolates.^19^ Several studies reported resistance to colistin among *A*. *baumannii* or *P. aeruginosa*.^19^, ^20^, ^21^ This difference in antibiotic resistance may be associated with the variation in the geographical area, clinical samples, and other factors. So, screening for the emergence of resistant strains is necessary for preventing the spread of resistant clones. TIG demonstrated considerable efficacy against *A. baumannii* isolates, and except for five isolates with intermediate resistance, the remaining were susceptible to TIG. Moreover, among *P. aeruginosa* isolates, 14 isolates were resistant to TIG. Elnasser et al. demonstrated that out of eight *A. baumannii* isolates, three showed resistance to tigecycline. Additionally, all 13 isolates of *P. aeruginosa* were found to be resistant to tigecycline.^22^ Although TIG has not been suggested by CLSI for antibiotic susceptibility testing of *A. baumannii* or *P. aeruginosa*, and the resistance rate was considerable among both species, we used TIG to improve the efficacy of MER and AMK against antibiotic‐resistant *A. baumannii* and *P. aeruginosa* isolates. While TIG had a synergistic effect with MER against 20 (80%) *A. baumannii* isolates, this effect was detected in only 5 (20%) *P. aeruginosa* isolates. It seems that the higher TIG resistance of *P. aeruginosa* isolates limits the efficacy of the MER‐TIG combination. Ju et al. demonstrated that combining colistin with ceftolozane/tazobactam in a two‐drug combination could serve as a valuable treatment option for infections caused by carbapenem‐resistant *A. baumannii*.^23^ Another study demonstrated that there was a synergistic effect between tigecycline and levofloxacin, amikacin, imipenem, and colistin against strains of *A. baumannii* that were not susceptible to tigecycline.^23^ Tsala et al. found that the combination of meropenem, tigecycline, and colistin demonstrated bactericidal activity against carbapenemase‐producing *K. pneumoniae* (CP‐Kp) isolates that were highly resistant.^24^ On the other hand, the relative susceptibility of *A. baumannii* to TIG suggests this antibiotic as a treatment option, and a proper synergy between MER and TIG can also make MER a therapeutic option in combination with TIG. In the current study, we also assessed the TIG‐AMK combination for a synergistic effect, which was observed in 10 (40%) *A. baumannii* isolates and none of the *P. aeruginosa* isolates. According to AST test results, *P. aeruginosa* isolates had higher TIG MICs than *A. baumannii*, and the lower rates of TIG‐AMK synergism probably are related to this issue. The synergistic effect of TIG‐AMK has also been reported for other bacterial species such as *K. pneumoniae* and non‐tuberculosis mycobacterium.^25^, ^26^ However, Moland et al. did not find TIG‐AMK synergism against *A. baumannii*.^27^ In contrast, Petersen et al. demonstrated the synergism of TIG with AMK in 45% of *A. baumannii* isolates, but not *P. aeruginosa* strains.^28^ Conversely, Principe et al. reported TIG‐AMK synergism in two (8.3%) *A. baumannii* isolates.^29^ Altogether, TIG‐AMK synergism is not a general phenomenon and depends on antibiotic susceptibility patterns and the origin of the isolates. MER‐IMI was another combination assessed in this study for a synergistic effect, which was observed in only three (12%) and five (20%) *A. baumannii* and *P. aeruginosa* isolates, respectively. The synergistic effect of double‐carbapenem combination has been reported against *K. pneumoniae* and *P. aeruginosa*.^30^ Although a previous study showed a considerable decrease in MICs of MER in combination with doripenem,^31^ the low frequency of significant amounts of FICI in our study demonstrates that using double‐carbapenem combination as a treatment choice for carbapenem‐resistant Gram‐negative bacteria is not always appropriate. However, FICI amounts of MER‐IMI were significant in isolates lacking MBL enzymes. Hence, double‐carbapenem combination can be considered for resistant isolated harboring resistance mechanisms other than MBL enzymes. The last combination studied herein was COL‐MER; 21 (84%)*A. baumannii* and 17 (68%) *P. aeruginosa* isolates had FICI ≤ 0.5. Using monotherapy instead of combination therapy may reduce the occurrence of adverse side effects associated with additional antibiotics; it is not recommended in the case of colistin. Several studies have demonstrated that administering colistin as a standalone treatment can result in the development of heteroresistant strains of *Acinetobacter* and *Pseudomonas*. Furthermore, it can lead to the emergence of colistin resistance among other isolates and promote the proliferation of organisms that are naturally resistant to colistin. This can ultimately lead to secondary bacterial infections, leaving clinicians with limited or no alternative treatment options combination of colistin with other antibiotics has been used against different Gram‐negative bacterial species, and most studies have reported considerable synergetic effects for different combinations of this antibiotic.^32^, ^33^, ^34^, ^35^ A meta‐analysis conducted^16^ indicated that combining colistin with a carbapenem, such as meropenem, effectively inhibited the development of colistin resistance and exhibited a synergy rate of over 50% against strains that were resistant to colistin. As a result, combination therapy was proposed as a potential approach to mitigate the emergence of resistance. The results of our study were consistent with a previous study conducted by Shields et al.,^36^ which investigated the effectiveness of colistin‐based antibiotic combinations against extensively drug‐resistant (XDR) *Acinetobacter* in laboratory settings. Both studies demonstrated positive interactions when colistin was combined with a carbapenem. The suggested mechanism for this synergistic effect involves the combined action of the two molecules on bacterial cells. Colistin works by targeting the outer membrane of the bacteria, causing changes in its permeability.^37^ This alteration allows meropenem to penetrate the bacteria at higher concentrations.^38^ The increased concentration of meropenem in the periplasmic space can then potentially suppress bacterial resistance mechanisms, making meropenem effective against resistant bacteria.^39^

## 5 CONCLUSION

Our results suggest that MER‐IMI synergy is more frequent against MBL‐lacking isolates, and this synergistic activity is lower against IMP, NDM, and VIM producers. On the other hand, higher MIC values of MER and IMI are related to lower FICI values. However, the benefit of the TIG‐MER and COL‐MER combinations against carbapenem‐resistant *A. baumannii* and *P. aeruginosa* isolates is independent of the β‐lactamase type. It is speculated that the aforementioned combinations could be promising options against carbapenem bacteria. However, further investigations are needed to confirm this claim.

## AUTHOR CONTRIBUTIONS

**Hossein Kazemian**: Conceptualization; investigation; validation; visualization. **Morteza Karami‐Zarandi**: Conceptualization; investigation. **Hamid Heidari**: Methodology; visualization; writing—original draft; writing—review and editing. **Roya Ghanavati**: Conceptualization; supervision; validation; writing—original draft. **Saeed Khoshnood**: Conceptualization; investigation; validation.

## CONFLICT OF INTEREST STATEMENT

The authors declare no conflict of interest.

## TRANSPARENCY STATEMENT

The lead author Roya Ghanavati, Saeed Khoshnood affirms that this manuscript is an honest, accurate, and transparent account of the study being reported; that no important aspects of the study have been omitted; and that any discrepancies from the study as planned (and, if relevant, registered) have been explained.

## Supporting information

Supporting information.

## Data Availability

The authors confirm that the data supporting the findings of this study are available within the article.
