# Supplementary material for: Antimicrobial combination effects against multidrug‐resistant Acinetobacter baumannii and Pseudomonas aeruginosa strains: A cross‐sectional study
Source: Health Sci Rep. 2024 Apr 29;7(5):e2061. doi: 10.1002/hsr2.2061 (PMC11058283; doi:10.1002/hsr2.2061)
Supplement: Supplementary file 1 — Supporting information. [file HSR2-7-e2061-s001.docx]

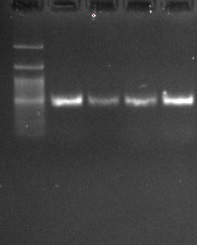


Figure 1. Gel electrophoresis image of  bla_OXA_*_-_*_48_ gene amplification (438 b.p.)


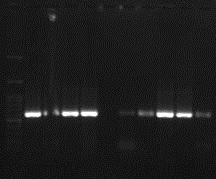


Figure 2. Gel electrophoresis image of  bla_OXA_*_-_*_58_ gene amplification (599 b.p.)


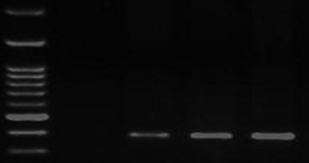


Figure 3. Gel electrophoresis image of  bla_OXA_*_-_*_23_ gene amplification (355 b.p.)


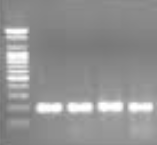


Figure 4. Gel electrophoresis image of bla_IMP_ gene amplification (194 b.p.)


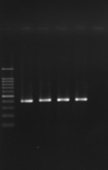


Figure 5. Gel electrophoresis image of bla_VIM_ gene amplification (390 b.p.)


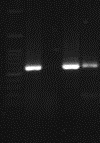


Figure 6. Gel electrophoresis image of bla_NDM_ gene amplification (621 b.p.)
